# *Neogenin* expression is inversely associated with breast cancer grade in *ex vivo*

**DOI:** 10.1186/1477-7819-12-352

**Published:** 2014-11-22

**Authors:** Wanying Xing, Qiang Li, Rangjuan Cao, Zheli Xu

**Affiliations:** Department of Breast Surgery, China-Japan Union Hospital of Jilin University, Changchun, 130033 China; Department of Hand Surgery, China-Japan Union Hospital of Jilin University, Changchun, 130033 China

**Keywords:** *Neogenin*, breast cancer, histological grading, predictive biomarkers

## Abstract

**Background:**

*Neogenin* is closely related to the human tumor suppressor gene deleted in colorectal cancer and plays a role in mammary morphogenesis. This study aimed to assess *neogenin* expression in breast cancer for any clinically significant association.

**Methods:**

A total of 54 breast cancer patients who underwent modified radical mastectomy were enrolled for immunohistochemical and quantitative real-time PCR analysis of *neogenin* expression in their cancerous breast tissues in comparison to matching distant non-cancerous tissues.

**Results:**

The data showed that the *neogenin* protein was either strongly or moderately expressed in the cytoplasm of the distant non-cancerous cells. In contrast, *neogenin* protein was either weakly or not expressed in the cytoplasm of 51/54 (94.4%) breast cancer cells, among which 13 breast cancer cases did not express *neogenin* protein at all (13/54, 24.1%). Similarly, levels of *neogenin* mRNA were significantly lower in breast cancer tissues than that of the matched distant non-cancerous tissues (51/54, 94.4%). *Neogenin* expression was inversely associated with breast cancer grade; that is, grade III breast cancer expressed much less *neogenin* than grade I-II (*P* < 0.05).

**Conclusions:**

This study indicates that *neogenin* expression in breast cancer tissues is inversely associated with tumor grade.

## Background

Breast cancer is the most common cancer in women in both developed and developing countries [1]. In China, breast cancer accounts for 14.2% of all malignant tumors in Chinese women, while the percentage is 26.4% in the USA [2]. Despite advancements in early detection and treatment of breast cancer, breast cancer is still the leading cause of cancer-related death among women in the world [3, 4]. Clinically, tumor stage, histological grade, and different tumor markers have been useful in evaluating and predicting breast cancer progression, treatment response, and prognosis [5]. Recently, researchers have tried to classify breast cancer based on the profile of differential gene expression to advance individualized treatment, and to help predict prognosis of the patients [6]. For example, breast cancer with estrogen receptor (ER) and progesterone receptor (PR) expression has been associated with sensitivity to endocrine therapy [7, 8], whereas human epidermal growth factor-2 (HER2)-overexpressed or HER-2-amplified breast cancer is resistant to endocrine therapy [9], but is more sensitive to trastuzumab [10, 11]. ER, PR and HER-2-negative breast cancer (that is, triple negative breast cancer) [12] has the worst prognosis among all subtypes of breast cancer. To date, early detection is still key for survival of patients. Thus, identification and evaluation of novel tumor markers could help with early detection of breast cancer or the development of novel therapeutic targets for treatment of breast cancer patients.

Towards this end, our study focused on the protein *neogenin*, which is closely related to the human tumor suppressor gene deleted in colorectal cancer (DCC) [12] and which plays a role in mammary morphogenesis [13]. *Neogenin* has been shown to be expressed in a wide range of tissues in vertebrates, especially in sites where cells actively proliferate and migrate. *Neogenin* ligands include netrins and the family of repulsive guidance molecules (RGM) [14–16]. *Neogenin* has various functions dependent on its activation by different ligands; for example, after binding to netrin-1, *neogenin* can improve axon guidance by chemoattractively promoting cell migration and adhesion, whereas when binding RGM?, *neogenin* functions as a chemorepellant for cells [17]. Overall, the *neogenin*-ligand interaction can influence cell migration [17], tissue morphogenesis [18, 19], tumor growth [20, 21] and regulation of inflammation [22]. However, *neogenin* can induce apoptosis of certain types of cells when its ligands are absent. In the mammary gland, it has been shown that netrin-1-activated *neogenin* can stabilize multipotent progenitor cap cells during mammary gland morphogenesis [22, 23], while another study showed that *neogenin* expression was inversely associated with mammary gland tumorigenesis [23]. However, in the original gene cloning and screening study [13], there was no alteration in *neogenin* expression observed in more than 50 types of human cancer cell lines, including breast cancer. In this study, we measured expression of *neogenin* mRNA and protein in breast cancer and compared this to expression in distant non-cancerous tissues in order to establish whether this receptor is clinically associated with breast cancer.

## Methods

### Breast cancer tissue samples

In this study, we recruited a total of 54 female primary breast cancer patients with a mean age of 51 years (range 30 to 75 years of age) who underwent modified radical mastectomy at the China-Japan Union Hospital of Jilin University between June 2012 and February 2013. None of these patients received preoperative chemo-, radiation-, or endocrine therapy. This study was approved by The Ethics Committee of Jilin University and all patients provided informed consent. Tissue specimens of breast cancer lesions and distant normal mammary glands were collected during the surgery, snap-frozen in liquid nitrogen, and stored at −80°C before use. Tumors were diagnosed and classified according to the American Joint Committee on Cancer breast cancer TNM staging system [24] and the World Health Organization breast cancer histology and subtypes classifications [25].

### Immunohistochemical staining

Formalin-fixed and paraffin-embedded tissue blocks were cut into 5 μm thick tissue sections for immunohistochemical staining of ER, PR, HER2, Ki67, p53, and *neogenin*. Tissue sections were deparaffinized and re-hydrated routinely and then subjected to antigen retrieval by boiling in a pressure cooker in 10 mM citrate buffer, pH 6.0 at a pressure of 0.12 MPa for 90 seconds. After treatment with 3% H_2_O_2_ for 30 minutes, the sections were incubated with 20% normal serum for 50 minutes and then with the primary antibody overnight at 4°C. The antibodies against ER, PR, HER2, Ki67, p53, and *neogenin* were obtained from Santa Cruz Biotechnology (Santa Cruz, CA, USA) and diluted according to the manufacturer’s instructions. On the following day, the sections were washed with PBS thrice and then processed using an ultrasensitive TM S-P kit (Maixin Biotechnology, Fuzhou, China). After washes in PBS, the color reaction was developed using a 3,3'-diaminobenzidine kit (Maixin Biotechnology). The sections were counterstained with hematoxylin and covered with a coverslip.

The stained tissue sections were reviewed and scored independently by two pathologists (Drs Yang Hua and Chen Guiqiu). The proportion of tumor cells was scored as follows: none (no positive tumor cells), weak (<10% positive tumor cells), moderate (10 to 50% positive tumor cells), and strong (>50% positive tumor cells). ER and PR positivity was defined as strong nuclear staining in at least 3/8 of the tumor cells reviewed. HER2/neu positivity was defined as strong (3+) membranous staining in at least 10% of tumor cells, whereas scores of 0 to 2+ were regarded as negative.

### RNA isolation, reverse transcription, and quantitative real-time PCR

Total cellular RNA was isolated from frozen tissues using Trizol reagent (Invitrogen, Carlsbad, CA, USA) according to the manufacturer’s protocol and was then incubated with DNase I (Invitrogen) to remove potentially contaminating genomic DNA. After purification, these RNA samples (5 μg each) were subjected to cDNA synthesis using an M-MLV reverse transcriptase kit (Promega, Madison, WI, USA). Next, these cDNA samples underwent quantitative PCR amplification using a StepOnePlus™ Real-Time PCR System (TaKaRa, Dalian, China). Primers for *neogenin* were 5'-ACA TGC TGC ACT GAT CAC CA-3' and 3'-TCA TAG GTG GGA GGT CCT GG-5'; for GAPDH were 5'-TGA TGA CAT CAA GAA GGT GGT GAA G-3' and 5'-TCC TTG GAG GCC ATG TGG GCC AT-3'. PCR conditions were set as follows: denaturation at 95°C for 5 minutes, followed by 40 cycles of 95°C for 5 seconds, 60°C for 10 seconds, and 72°C for 30 seconds, and an additional cycle at 85°C for 30 seconds to measure the SYBR Green fluorescence. Finally, the melting-curve was generated by slowly heating the PCR reactions to 95°C (by 0.3°C per cycle) while simultaneously measuring SYBR Green signal intensity. Relative mRNA expression of *neogenin* in all the tissue samples was normalized to that of GAPDH using the equation of 2^−∆∆ CT^.

### Statistical analysis

SPSS18.0 software (SPSS, Chicago, IL, USA) was used to statistically assess the *P* value. Measurement data were analyzed by Student’s *t*-test, while categorical data were analyzed by the chi-square test. Differences were considered statistically significant at *P* < 0.05.

## Results

### Differential expression of *neogenin*in breast cancer and its distant non-cancerous tissues

**Figure 1:**
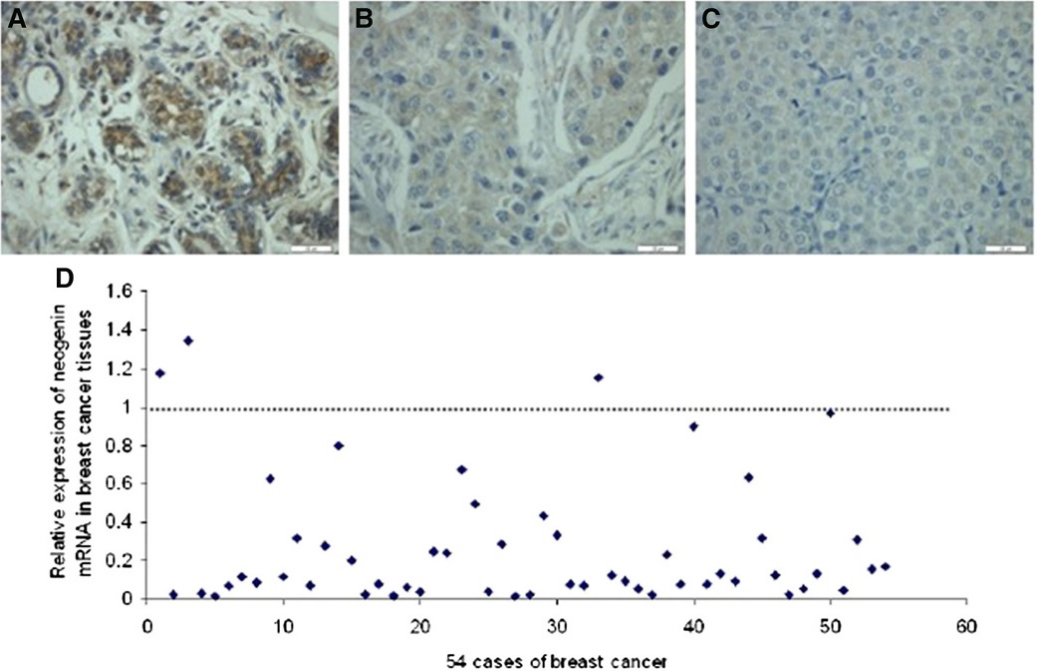
**Differential expression of** ***neogenin*** **mRNA and protein in breast cancer and in matching distant non-malignant tissues. (A)** Immunohistochemistry; a representative case showing strong expression of *neogenin* protein in the cytoplasm of distant non-cancerous tissue. 200× magnification. **(B)** Immunohistochemistry; a representative case showing weak expression of *neogenin* in the cytoplasm of breast cancer tissues (n = 51). 200× magnification. **(C)** Immunohistochemistry; a representative case showing negative expression of *neogenin* protein in breast cancer tissue (n = 13). **(D)** Quantitative real-time PCR; 51 out of 54 cases of breast cancer tissue showed a low level of *neogenin* mRNA compared to that of the matching distant non-cancerous tissue. The level of *neogenin* expressed in each distant non-cancerous tissue was normalized to 1 as a control.

We first detected expression of *neogenin* protein using immunohistochemical staining in 54 breast cancer and distant normal tissue samples. The results were scored as none, weak, moderate, and strong *neogenin* staining of epithelial cells according to the assessment of two independent pathologists. *Neogenin* protein was strongly or moderately expressed in the cytoplasm of the distant non-cancerous cells (Figure 1A). In contrast, *neogenin* protein was weakly or not stained in the cytoplasm of breast cancer cells (51/54, 94.4%; Figure 1B), among which 13 breast cancer cases did not express *neogenin* protein at all. (13/54, 24.1%; Figure 1C).

We then assessed *neogenin* mRNA expression in these tissue samples and found that the level of *neogenin* mRNA was significantly lower in breast cancer tissues than in the matched distant non-cancerous tissues (51/54, 94.4%; Figure 1D).

### Association of *neogenin*expression with clinicopathological parameters from breast cancer patients

**Figure 2:**
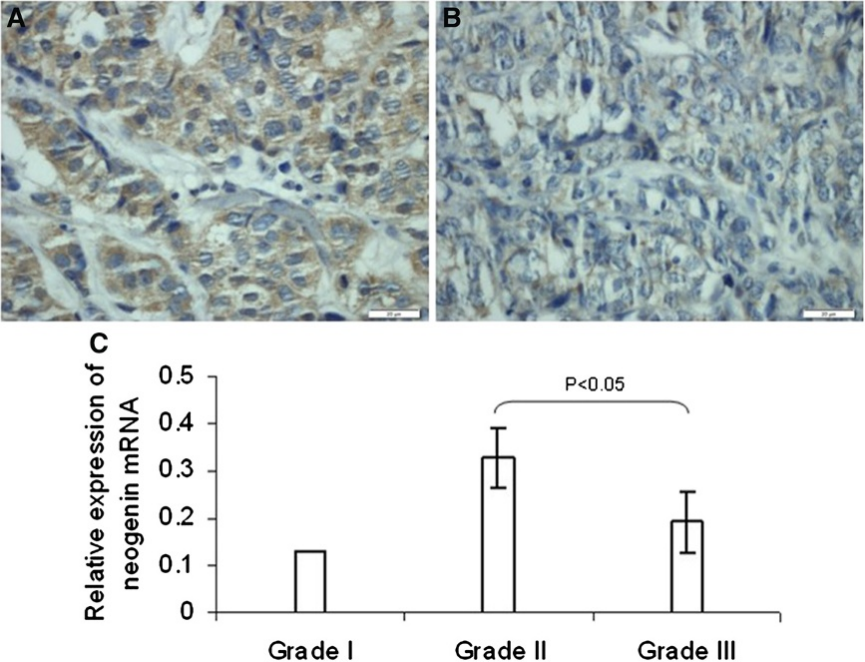
**Association between** ***neogenin*** **expression and clinicopathological parameters from breast cancer patients. (A)** Expression of *neogenin* in histology grade II breast cancer tissue (n = 29). 200× magnification. **(B)** *Neogenin* expression in histology grade III breast cancer tissue (n = 24). 200× magnification. **(C)** Expression of *neogenin* mRNA in different histological grades of breast cancer.

We then associated the expression of *neogenin* protein with clinicopathological parameters from breast cancer patients (Table 1). The results show that *neogenin* expression is inversely associated with breast cancer grade; that is, grade III breast cancer expressed less *neogenin* than grade I-II (Figure 2A,B). Similar findings were observed with regard to *neogenin* mRNA expression (*P* < 0.05; Figure 2C). However, there was no association between *neogenin* expression and other clinicopathological parameters, such as tumor size, lymph node status, vascular invasion status, breast cancer subtype, TNM stage, and biomarker (ER, PR, HER-2, and Ki67).

**Table 1:** **Association between** ***neogenin*** **mRNA and protein levels and clinicopathological factors from breast cancer**

Clinicopathological features	Variable			***P***value
		N (%)	Expression of ***neogenin***mRNA level mean ± SD	
Age (years)	≥65	4 (7.4)	0.042 ± 0.035	0.060
	<65	50 (92.6)	0.270 ± 0.323	
Tumor size (cm)	≤2.00	24 (44.4)	0.298 ± 0.373	0.959
	2.01-5.00	26 (48.1)	0.217 ± 0.280	
	≥5.00	4 (7.4)	0.372 ± 0.412	
Histology grade	I	1 (1.9)	0.132 ± 0	0.032
	II	29 (53.7)	0.330 ± 0.343	
	III	24 (44.4)	0.192 ± 0.313	
Stage	I	12 (22.2)	0.206 ± 0.319	0.818
	II	19 (35.2)	0.292 ± 0.322	
	III	14 (25.9)	0.254 ± 0.357	
	IV	9 (16.7)	0.302 ± 0.368	
Lymph node metastasis	Yes	35 (64.8)	0.320 ± 0.356	0.16
	No	19 (35.2)	0.164 ± 0.257	
Vascular invasion	Yes	38 (70.4)	0.272 ± 0.357	0.755
	No	16 (29.6)	0.247 ± 0.268	
Subtype	Luminal A	13 (24.1)	0.407 ± 0.400	0.484
	Luminal B	14 (25.9)	0.217 ± 0.314	
	H type	14 (25.9)	0.199 ± 0.236	
	TNBC	13 (24.1)	0.244 ± 0.356	
ER	+	27 (50)	0.309 ± 0.364	0.387
	-	27 (50)	0.221 ± 0.295	
PR	+	21 (38.9)	0.279 ± 0.318	0.908
	-	33 (61.1)	0.256 ± 0.344	
HER2	+	21 (38.9)	0.239 ± 0.290	0.918
	-	33 (61.1)	0.281 ± 0.358	
Ki67	+	39 (72.2)	0.256 ± 0.333	0.128
	-	15 (27.8)	0.287 ± 0.335	
p53	+	21 (38.9)	0.308 ± 0.371	0.292
	-	33 (61.1)	0.237 ± 0.305	

ER, estrogen receptor, HER-2, human epidermal growth factor-2; PR, progesterone receptor; TNBC, triple negative breast cancer.

## Discussion

To date, *neogenin* expression has been shown to be downregulated in a variety of human cancers such as glioblastoma [26], colon cancer [27], prostate cancer [28], and breast cancer [24]. In the original *neogenin* cloning and screening study, Meyerhardt and colleagues [13] reported no alteration in *neogenin* expression in more than 50 different human cancer cell lines, including breast cancer cell lines. Later, Lee and colleagues [23] measured expression of *neogenin* in breast cancer cell lines and in eight matched breast cancer and adjacent non-cancerous tissues using Western blot, and in only breast cancer tissues by using tissue array. They found that *neogenin* expression was downregulated in both breast cancer cell lines as well as cancerous tissues and concluded that *neogenin* expression was inversely correlated with mammary carcinogenicity. Our current data support this and other previously published data [24] in that we have found an association between *neogenin* expression and breast cancer grade. We observed no *neogenin* expression in higher histological grade breast cancer compared to lower grade tumors, which is consistent with a study of glioma [22] where *neogenin* expression was inversely associated with histological grade of that cancer. *Neogenin* expression has been reported to be even lower in recurrent glioma cases compared to that of their primary tumors [22]. The histological grading system in breast cancer is based on differentiation of tumor cells, which is an important factor in predicting prognosis of breast cancer patients and tumor aggressiveness. Thus, we speculate that breast cancer with lower *neogenin* expression in the high histological grade might be more likely to recur and/or have a worse prognosis. However, the majority of our patients had grade II and III breast cancers which precluded precise evaluation of *neogenin* expression in grade I breast cancer. Future studies should recruit patients with this breast cancer grade to further confirm that *neogenin* expression is associated with breast cancer grade.

In the current study, we observed that level of *neogenin* expression was particularly low in the four patients who were 65 years or older. In parallel with our findings, Bondy and colleagues [29] also showed that minimum levels of *neogenin* appeared in older glioma patients with poor prognosis. As older patients frequently have systemic diseases after being diagnosed with breast cancer, treatment of older patients is conservative compared to younger ones [30]. Thus, *neogenin* should be further evaluated as a potential biomarker for older breast cancer. Based on our clinical experiences, malignancy of breast cancer is closely associated with tumor size, subtype, TNM stage, lymph node metastasis, vascular invasion, and expression of other biomarkers (such as ER, PR HER-2, and Ki67); however, we failed to find any statistical significance between *neogenin* expression and these prognostic factors. This indicates that *neogenin* expression and its functions warrant further investigation in breast cancer. A previous study showed that the function of *neogenin* in normal breast development is to guide cap cells and luminal cells into juxtaposition during the adolescent development period [22]. Although the combination of netrin-1 and *neogenin* plays a significant role during this process, the role of *neogenin* in breast cancer remains to be determined. It has previously been shown that *neogenin* might function as an independent receptor in breast cancer to suppress tumor development [24]. When *neogenin* levels are downregulated, *neogenin*-induced apoptosis could be interrupted which could, in turn, lead to cancer development [20, 31]. Similarly, other studies suggest that *neogenin* could induce chemoattraction or chemorepulsion after binding to ligands such as netrin-1 and RGM to influence tumor cell migration and invasion [21, 32]. In addition, we have shown in the current study that *neogenin* expression is only marginally associated with lymph node metastasis of breast cancer.

## Conclusions

As such, we hypothesize that the level of *neogenin* expression could be altered during tumorigenesis and that a lack of *neogenin* expression could promote tumorigenesis early in the process but that, following tumor formation, certain tumor cells may re-express *neogenin* protein to promote tumor cell migration and metastasis. However, further studies are needed to clarify the function of *neogenin* as well as the cause of lost *neogenin* expression in breast cancer before *neogenin* can be established as a biomarker for breast cancer diagnosis.

## Consent

Written informed consent was obtained from the patient for the publication of this report and any accompanying images.

## Abbreviations

DCC: deleted in colorectal cancer
ER: estrogen receptor
HER-2: human epidermal growth factor-2
PBS: phosphate-buffered saline
PCR: polymerase chain reaction
PR: progesterone receptor
RGM: repulsive guidance molecules.
